# Coherence Function and Adaptive Noise Cancellation Performance of an Acoustic Sensor System for Use in Detecting Coronary Artery Disease

**DOI:** 10.3390/s22176591

**Published:** 2022-08-31

**Authors:** Matthew Fynn, Sven Nordholm, Yue Rong

**Affiliations:** School of Electrical Engineering, Computing and Mathematical Sciences (EECMS), Faculty of Science and Engineering, Curtin University, Bentley, WA 6102, Australia

**Keywords:** coherence function, adaptive noise cancellation, Wiener filter, coronary artery disease

## Abstract

Adaptive noise cancellation is a useful linear technique to attenuate unwanted background noise that cannot be removed using traditional frequency-selective filters. Usually, this is due to the signal and noise co-existing in the same frequency band. This paper tests a weighted least mean squares (WLMS) algorithm on a stethoscope system for use in detecting coronary artery disease in the presence of background noise. Each stethoscope is equipped with two microphones: one used to detect heart signals and one used to detect background noise. The WLMS method was used for four different sources of background noise whilst measuring a heartbeat, including a single tone, multiple tones, hospital/clinic noise, and breathing noise. The magnitude-squared coherence between both microphones was unity for the tone scenarios, resulting in complete attenuation. For the other background noise sources, a less-than-unity magnitude-squared coherence resulted in minor and no attenuation. Thus, the coherence function is a tool that can be used to predict the amount of attenuation achievable by linear adaptive noise-cancellation techniques, such as WLMS, as presented in this article.

## 1. Introduction

Heart auscultation—the interpretation of heart sounds by a physician—is a common method utilised for cardiac diagnosis [1]. Many physicians are documented to have poor auscultatory skills due to their difficulty to acquire, yet it remains the primary method used for diagnosis and screening in primary healthcare [2,3,4]. The leading cause of mortality and morbidity in the world is cardiovascular disease, contributing 31% towards all global deaths in 2019 according to the World Health Organisation [5]. This is particularly worrisome for low-to-middle-income countries where high-quality diagnostics are difficult to obtain [6]. To address this large and complex problem, there is a global need for the affordable pre-screening of coronary artery disease (CAD).

Data quality will be a limiting factor in the achievable accuracy of any classification technique of CAD. Signal corruption from unwanted noise will limit data quality. In the available public datasets of normal and abnormal heartbeats, noise limits the accuracy achievable by machine learning and classifiers [7], with frequency-selective filtering being the only technique used to reduce noise. Thus, cancelling noise in the frequency band of interest is a crucial stage in acquiring data and has been implemented in the field of biomedical signals [8,9,10]. A comprehensive review of adaptive filters for noise cancellation in [11] identified existing methods derived from the least mean squares (LMS) method, highlighting it as a preferred method for noise cancellation due to its robustness and simplicity. This study shows that these techniques can be implemented on a digital stethoscope with background-noise-sensing capability and is preliminary to future classification studies. A weighted least mean squares (WLMS) adaptive filter algorithm is implemented in this study.

**Figure 1:**
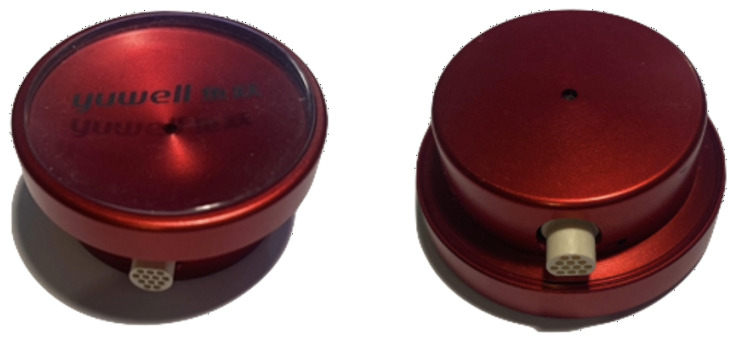
Single stethoscope. HM facing upward (**left**) and BNM facing upward (**right**).

A system has been developed that incorporates six digital stethoscopes onto a wearable vest that simultaneously measures heartbeat signals and uses machine learning methodologies to pre-screen for CAD. Each stethoscope, shown in Figure 1, has two microphones. The first microphone is the heart-sensor microphone (HM) and is located behind the diaphragm. The second microphone is the background-noise microphone (BNM) located at the other end of the stethoscope. The assumption is that the HM will pick up the heart signal plus part of the background noise, and that the BNM will pick up just the background noise. Using the two-microphone configuration, it is hypothesised that the background noise can be reduced from the heart-sensor microphone. This will contribute to successful diagnosis techniques, as acquired signals from the system will be cleaner with higher signal integrity. The coherence function is the measure of the causal relationship between two signals with the presence of other signals [12] and is utilised in this paper to predict the attenuation of the background noise in the HM signal. Using a single tone whilst measuring a heartbeat, we tuned a WLMS adaptive filter algorithm to achieve complete attenuation from the HM. The parameters from this exercise were then used in this study and tested on other noise sources.

## 2. Background

Background noise can couple into the HM and corrupt acoustic heartbeat measurements. This can decrease signal integrity, as it cannot be filtered out using conventional frequency-selective filters if the noise lies within the frequency band of interest of 25–250 Hz [13]. To attenuate unwanted background noise or enhance the desired signal, adaptive filtering techniques and other non-linear techniques can be utilised.

### 2.1. Adaptive Noise Cancellation

Noise cancellation via a Wiener filter is a linear process, where the data are assumed to be linear-time-invariant (LTI). As explained in [14], a (*p* − 1)st-order Wiener filter, given by Equation (1), can be derived, which produces the minimum mean-square estimate (MMSE) of a given process d(n) by filtering a statically related process x(n) that has unwanted noise. The output of the filter $\hat{d}\left(n\right)$ is the convolution of w(n) and x(n) given by Equation (2).

(1) $$W\left(z\right)=\sum_{n=0}^{p-1}w\left(n\right)z^{-n}$$

(2) $$\hat{d}\left(n\right)=\sum_{l=0}^{p-1}w\left(l\right)x\left(n-1\right)$$

Here, we assume that the auto-correlation functions $r_{x}\left(k\right)=E\left[x\left(n\right)x\left(n+k\right)\right]$ and $r_{d}\left(k\right)=E\left[d\left(n\right)d\left(n+k\right)\right]$ and the cross-correlation $r_{dx}\left(k\right)=E\left[d\left(n\right)x\left(n+k\right)\right]$ are known. The Wiener coefficients are calculated to minimize the mean-square error given by Equation (3).

(3) $${E\{|e\left(n\right)|}^{2}\}=E\{|d\left(n\right)-\hat{d}\left(n\right){|}^{2}\}$$

**Figure 2:**
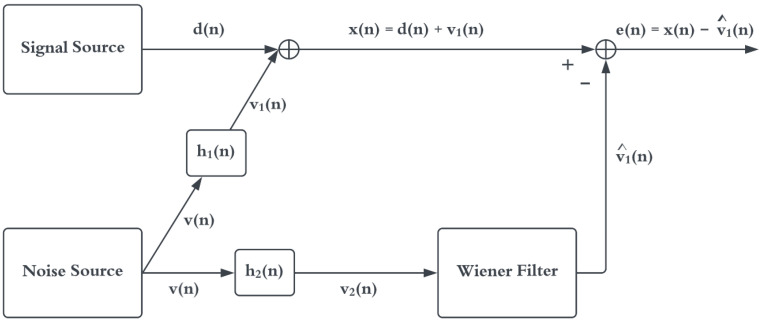
Using a secondary sensor for noise cancellation. Adapted from [14].

Minimizing Equation (3) derives the *Winer–Hopf equations*, shown in Equation (4):

(4) $$R_{x}w=r_{dx},$$

where w represents the filter coefficients, and $R_{x}$ is a p×p Hermitian Toeplitz matrix of auto-correlations [14]. A signal- and noise-cancellation model is shown in Figure 2. The desired heartbeat signal to be measured, d(n), is corrupted by various background noise sources, v(n), to produce the signal measured by the HM, $x\left(n\right)=d\left(n\right)+v_{1}\left(n\right)$. Without any information about $v_{1}\left(n\right)$, it is not possible to remove it from d(n). The BNM measures the noise sources directly, depicted as $v_{2}\left(n\right)$; however, the two sensors do not detect the noise the same way $\left(v_{1}\left(n\right)\neq v_{2}\left(n\right)\right)$, meaning the desired signal cannot be yielded via a direct subtraction [9]. Instead, a Wiener filter produces an estimate of $v_{1}\left(n\right)$, denoted as ${\hat{v}}_{1}\left(n\right)$, via the observational measurements of $v_{2}\left(n\right)$. This estimate is subtracted from x(n) to attenuate the background noise. Here, the Wiener–Hopf equations are given by Equation (5).

(5) $$R_{v_{2}}w=r_{xv_{2}}$$

**Figure 3:**
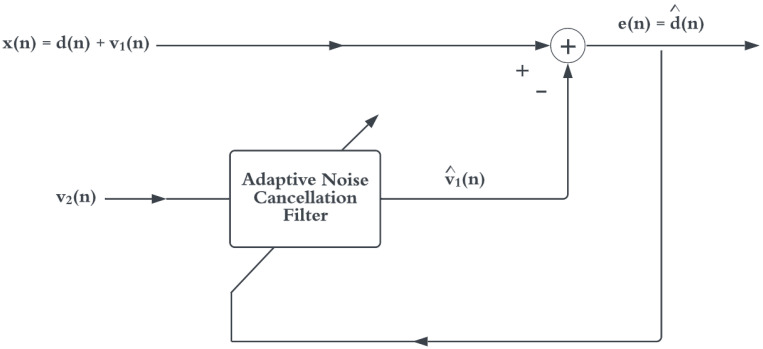
Adaptive noise canceller. Adapted from [14].

In environments where the noise source is constantly changing, adaptive filters are needed that update the filter coefficient w in Equation (5) in real time. An adaptive filter model is shown in Figure 3.

Here, the output of the adaptive filter aims to minimise the MSE of estimating $v_{1}\left(n\right)$; thus, e(n) is the MSE of the desired signal d(n). The optimum filter coefficients that minimise this error are found by solving Equation (5), which can be difficult, as they constantly change for each value of *n*. To simplify this requirement, the form of Equation (6) can be utilised to update the filter coefficients at each time *n*.

(6) $$w_{n+1}=w_{n}+\Delta w_{n}$$

Here, the filter coefficient w at time *n* is corrected by $\Delta w_{n}$ to form a new set of coefficients, $w_{n+1}$, at time n+1. Defining how this correction is to be applied is at the heart of adaptive algorithms.

### 2.2. Coherence Function

The coherence between the HM and BNM channels indicates how much noise can be attenuated at particular frequencies and is a measure of the accuracy between the assumed linear channels [12]. As derived in [15], the coherence function, sometimes referred to as the coherence-squared function, may be defined by Equation (7):

(7) $$\gamma_{vx}^{2}\left(f\right)=\frac{|S_{vx}{\left(f\right)|}^{2}}{S_{vv}\left(f\right)S_{xx}\left(f\right)},$$

where $S_{vx}\left(f\right)$ is the cross-spectral density, and $S_{vv}\left(f\right)$, $S_{xx}\left(f\right)$ are the auto spectral densities, which are assumed to exist. This is valid for all frequencies *f* and is bounded by the condition:

(8) $$0\leq \gamma_{vx}^{2}\left(f\right)\leq 1$$

If we apply this ideology to a noise-free LTI system governed by x(t)=v(t)∗h(t), where h(t) is the system impulse response, and ∗ denotes the convolution, or X(f)=V(f)H(f), where H(f) is the transfer function, the auto- and cross-spectral densities are governed by Equations (9) and (10):

(9) $$S_{xx}\left(f\right)={\left|H\left(f\right)\right|}^{2}S_{vv}\left(f\right)$$

(10) $$S_{vx}=H\left(f\right)S_{vv}\left(f\right)$$

**Figure 4:**
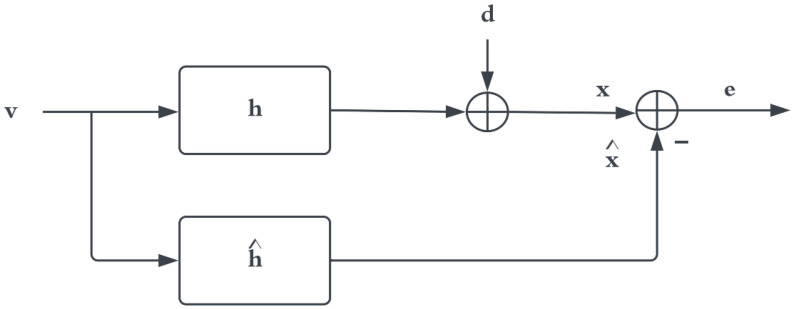
Linear system model.

Here, $\gamma_{vx}^{2}\left(f\right)=1$ and $S_{vv}\left(f\right)$ and $S_{xx}\left(f\right)\neq 0$ for any frequency. If a heartbeat signal is introduced in x(t) depicted as x(t)=v(t)∗h(t)+d(t), or if the system is non-linear, then $\gamma_{vx}^{2}\left(f\right)\leq 1$. We can model this system as a linear system, shown in Figure 4, to deduce if there is a linear relationship between v(t) and x(t).

The true system is modelled by Equation (11), and the model is obtained by minimising the error given by Equation (12).

(11) $$\hat{x}\left(t\right)=v\left(t\right)*\hat{h}\left(t\right)$$

(12) $$e\left(t\right)=x\left(t\right)-\hat{x}\left(t\right)$$

The MMSE method is a standard way to achieve this in the time or frequency domain. The frequency domain MMSE method is as follows.

(13) $$\begin{array}{cc} \min_{\hat{H^{*}}}R_{ee}\left(f\right) & =\lim_{T\to \infty }{\hat{E}}_{T}\left(X_{T}\left(f\right)-{\hat{X}}_{T}\left(f\right)\right)\\ \; & =R_{xx}\left(f\right)-R_{x\hat{x}}\left(f\right)-R_{\hat{x}x}\left(f\right)+R_{\hat{x}\hat{x}}\left(f\right)\\ \; & =R_{vv}\left(f\right){\left|H\left(f\right)\right|}^{2}+R_{dd}\left(f\right)-H\left(f\right)R_{vv}\left(f\right)\hat{H^{*}}\left(f\right)\\ & \;\;\;\;\;-\hat{H}\left(f\right)R_{vv}\left(f\right)H^{*}\left(f\right)+{\left|\hat{H}\left(f\right)\right|}^{2}R_{vv}\left(f\right) \end{array}$$

Here, $X_{T}\left(f\right)$ and ${\hat{X}}_{T}\left(f\right)$ are the frequency–domain representation of x(t) and $\hat{x}\left(t\right)$, respectively. Taking the derivative of Equation (13) with respect to $\hat{H^{*}}$ yields:

$$-H\left(f\right)R_{vv}+\hat{H}\left(f\right)R_{vv}\left(f\right)=0,$$

which leads to:

(14) $$\begin{array}{cc} \hat{H}\left(f\right) & =H(f), \end{array}$$

implying:

(15) $$\begin{array}{c} R_{ee}\left(f\right)=R_{dd}\left(f\right). \end{array}$$

**Figure 5:**
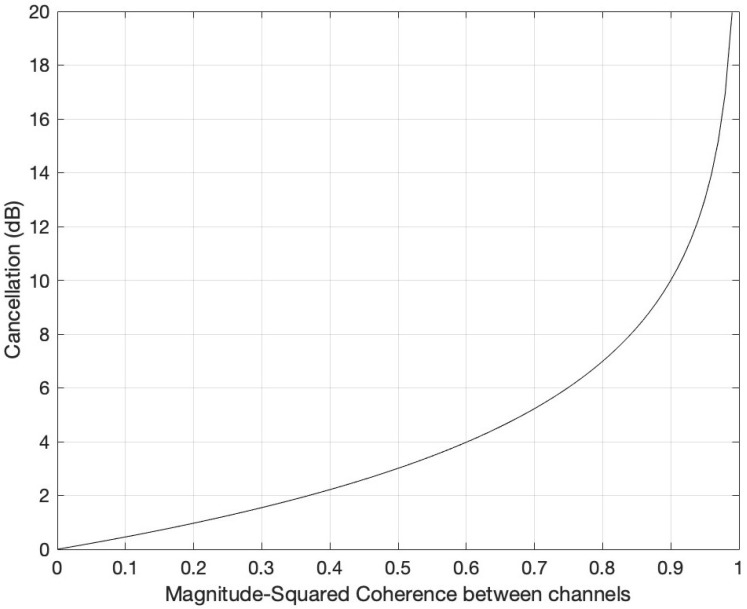
Adaptive cancellation vs. the squared coherence between $v_{2}$ and *x*.

Here, we assumed that E(dv)=0, resulting in $R_{dv}\left(f\right)=0$. We see that, for this scenario, the coherence function is given as:

(16) $$\begin{array}{cc} \gamma_{vx}^{2}\left(f\right) & =\frac{{\left|H\left(f\right)\right|}^{2}R_{vv}^{2}\left(f\right)}{R_{vv}\left(f\right)({\left|H\left(f\right)\right|}^{2}R_{vv}\left(f\right)+R_{dd}\left(f\right))}\\ \; & =\frac{1}{1+\frac{R_{dd}\left(f\right)}{{\left|H\left(f\right)\right|}^{2}R_{vv}\left(f\right)}}\\ \; & =\frac{1}{1+\frac{R_{dd}\left(f\right)}{R_{xx}\left(f\right)-R_{dd}\left(f\right)}}. \end{array}$$

thus,

(17) $$1-\gamma_{vx}^{2}\left(f\right)=\frac{R_{dd}}{R_{xx}},$$

which connects the LTI noise-cancellation approach to the coherence function. One of the major problems encountered when applying adaptive noise-cancellation techniques in real acoustic environments was the low coherence between the noise signal corrupting the desired signal and the noise sensor [16]. For the scenario depicted in Figure 2, a coherence value less than unity indicates that the system relating *x* and $v_{2}$ is not linear [15], thus the noise at those particular frequencies cannot be completely removed. The amount of possible noise cancellation for this scenario as a function of frequency, R(ω), is given by Equation (18) and plotted in Figure 5 [16].

(18) $$R\left(\omega \right)=\frac{1}{1-|\gamma_{v_{2}x}^{2}\left(\omega \right)|}$$

## 3. Literature Survey

An adaptive filter has four main aspects, as stated in [17]: the signal, the structure, the parameters, and the adaptive algorithm. The signal is processed by the filter, the structure relates the output signal computed from the input signal, the parameters alter the input–output relationship iteratively, and the adaptive algorithm describes how these parameters are changed over time. Adaptive noise cancellation is an application of adaptive filtering that has been studied vastly in the literature [11], with the theory being explained throughout Section 2. Research in this area dealing with bio-signals has grown over the recent years, in particular due to the filtering of EEG and ECG signals that are corrupted by line interference [18,19]. The following part of this research discusses studies that directly apply to enhancing bio-generated sounds in electronic stethoscopes.

Cancelling environmental noise from cardiac sounds was investigated by Giustina et al. using a multi-channel adaptive algorithm [9]. Sound acquired from two microphones was used to reconstruct the transfer function of a stethoscope head allowing distortionless noise reduction. The first microphone was placed in a pipe directly connected to the stethoscope head, and the second microphone was placed in an adjacent pipe. It was experimentally verified that the inner microphone detected cardiac sounds and noise and the outer microphone detected only noise. A weighted difference (WD) adaptive algorithm was implemented and resulted in significant noise-reduction ratios (NRR) for different scenarios. In particular, a noisy office with background voices, a synthetic chirp and a high-frequency noise obtained NRRs of −14.04, −15.5 and −16.11 dB, respectively.

A similar study was conducted by Patel et al. in [10], where the aim was to attenuate simulated USAF C-130 aircraft noise whilst measuring and processing lung sounds. A two-channel stethoscope was held to a subject’s right anterior upper chest while they breathed through a pneumotachograph. The two microphones, labelled *x* (background noise pickup microphone) and *d* (subject microphone), offer the same functionality as seen in [9] and the proposed stethoscope shown in Figure 1. The signals from *x* and *d* were amplified and high-pass filtered at a 100 Hz cut-off frequency, then digitized at 5120 samples per second. Some 20-s data segments were recorded during breathing for aircraft noise levels of 80, 90 and 100 dB sound pressure levels (SPL). These epochs were processed with the adaptive-noise-reduction least mean squares (LMS) and normalised least mean squares (NLMS) algorithms using MATLAB. Both of these algorithms apply Equation (6) in different ways and are explained comprehensively in [10,14]. The LMS approach achieved approximately 15 dB of noise reduction in the frequency range 100–600 Hz at 100 dB SPL. The NLMS algorithm provided faster convergence with frequencies above 450 Hz attenuated by an additional 5 dB.

## 4. System Model

This paper uses a weighted least mean squares (WLMS) algorithm to update the filter coefficients shown in Equation (6). Let the HM signal corrupted with noise be denoted as x and the BNM signal be denoted as y. For each sample from the HM, x(i), the error history is given by Equation (19):

(19) $$e\left(i\right)=x\left(i\right)-w^{T}y\left(i\right)$$

Here, $y\left(i\right)={\left(y\left[i\right],y\left[i+1\right],\ldots ,y\left[i+N\right]\right)}^{T}$, where N is the filter length (FL). The weights of the filter were updated for each sample as per Equation (20):

(20) $$w=\left(1-0.001\right)w+\mu \frac{y{\left(i\right)}^{*}e\left(i\right)}{{\left||y\left(i\right)|\right|}^{2}}$$

Here, the FL determines the size of vectors y and w, and the step size, μ, determines the size at which the filter coefficients are updated. This algorithm is a “variable leaky LMS” algorithm, which has the potential to significantly outperform the standard LMS algorithm [20].

## 5. Materials and Methods

**Figure 6:**
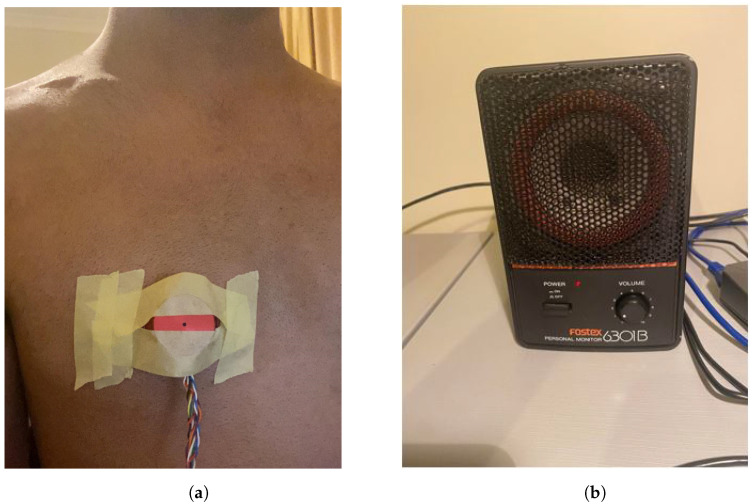
(**a**) Stethoscope taped to chest recording heartbeat with BNM exposed. (**b**) Background noise played through Fostex 6301B Speaker.

Four different sources of background noise were tested on heartbeat measurements: a single 300 Hz tone; multiple tones consisting of 200 Hz, 300 Hz and 500 Hz; hospital/clinic noise; and breathing noise. The tones were generated through Audacity, and the hospital/clinic noise was used from YouTube. The heartbeat measurements were of the first author and offer no diagnostic insight; thus, no ethical approval was required for this research. A FireFace UCX was used with a MATLAB interface to allow the simultaneous playback of the background noise through a Fostex 6301B speaker whilst recording from the stethoscope shown in Figure 1. The stethoscope was taped to the first author’s chest, making sure that the BNM was exposed. Figure 6 displays this configuration.

Heartbeat measurements with a duration of 15 s were taken for each background noise scenario. The first author was under breath-held conditions, except for the breathing noise scenario, where the speaker was turned off. The FireFace UCX collected data at 44.1 kHz sampling frequency, which was re-sampled down to 2 kHz. Version R2022a of MATLAB was used to complete this, as well as implement the algorithm from Section 4 on a laptop (HP laptop; Processor—Intel(R) Core(TM) i7-10510U; RAM—16 GB; Operating system—Windows 10; Processor Speed—2.3 GHz).

The FL and step size μ were first tuned on the single 300 Hz tone. The combination that achieved the best attenuated signal was then used for the other background noise scenarios after comparing the performance to a conventional LMS algorithm reviewed in [11]. For each case, the coherence function between the HM and the BNM was plotted via a Welch estimation in MATLAB to indicate the expected noise cancellation performance. Power spectral density (PSD) plots and spectrograms generated through MATLAB allowed visualisation of the performance.

## 6. Results and Discussion

This section consists of four subsections each containing the results from the four tested background noise sources; a single tone, multiple tones, hospital/clinic noise, and breathing noise. As the HM and BNM were re-sampled down to 2 kHz, the x-axis and y-axis range from 0–1 kHz for coherence function and spectrogram plots, respectively.

### 6.1. Single 300 Hz Tone

**Figure 7:**
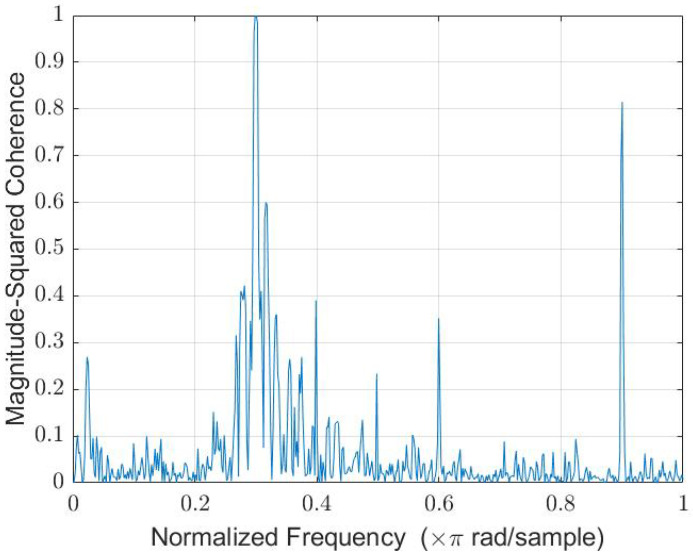
The 300 Hz tone background noise coherence function via Welch.

When a single 300 Hz tone was played through the Fostex speaker, the coherence function between the HM and BNM, shown in Figure 7, displayed unity at 300 Hz. Thus, we can expect complete attenuation of the tone using linear techniques.

**Figure 8:**
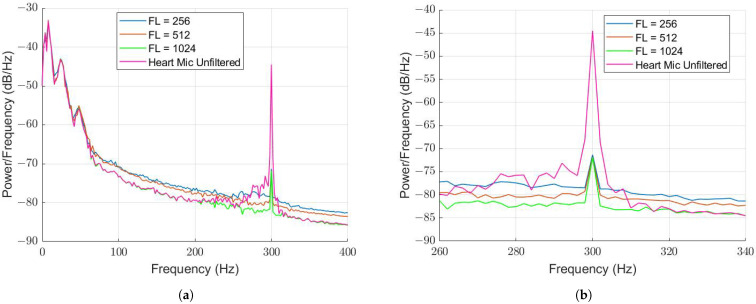
PSD comparison of different filter lengths with 300 Hz tone background noise and μ=0.5. (**a**) Comparison from 0–400 Hz. (**b**) Comparison from 260–340 Hz.

The WLMS algorithm was tested for FL = 256, 512 and 1024. The PSD of the filtered HM signals plotted with the unfiltered HM signal is displayed in Figure 8.

**Figure 9:**
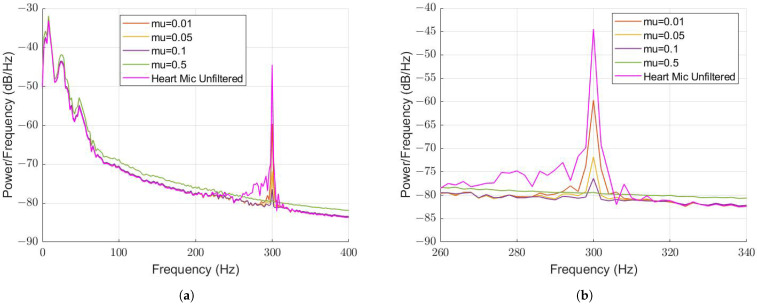
PSD comparison of different μ values with 300 Hz tone background noise and FL = 512 (**a**) Comparison from 0–400 Hz. (**b**) Comparison from 260–340 Hz.

For each scenario, the amount of attenuation remained constant at 27 dB. A FL of 512 was selected for tuning the step size value, μ, of the WLMS algorithm. Values of 0.01, 0.05, 0.1 and 0.5 were tested, with the PSDs compared once again in Figure 9.

The attenuation increased as μ increased, where almost complete attenuation (approx. 35 dB) was achieved at μ=0.5. However, we noted that the low frequency content was affected, evident in Figure 9a, which must be investigated further. Nevertheless, this agrees with the coherence function that suggests that complete attenuation is possible. A 300 Hz tone is a periodic signal, suggesting why the measured attenuation was high. This may not be the case when other non-periodic noise sources are present.

**Figure 10:**
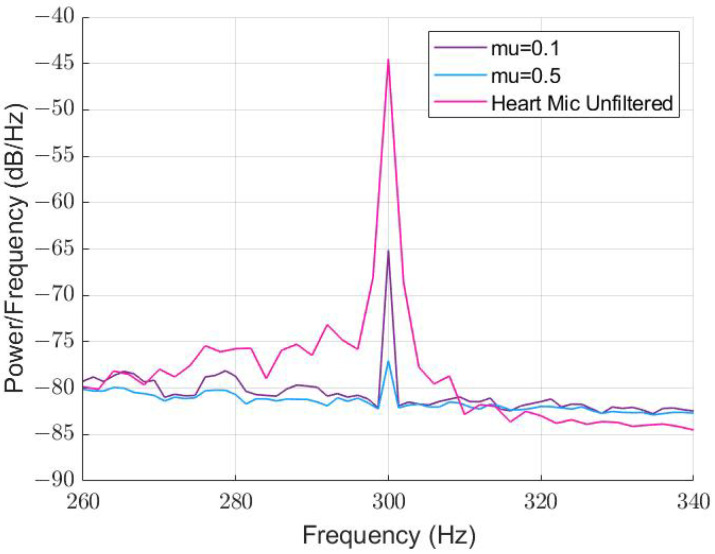
PSD comparison for different μ vales with 300 Hz tone background noise using a conventional LMS algorithm. FL = 512.

The performance using a conventional LMS algorithm is shown in Figure 10 based on the highest-performing parameters achieved in the WLMS algorithm. The filter length was kept at 512, and the algorithm tested for μ values of 0.1 and 0.5. The maximum attenuation achieved was 32 dB, when μ was 0.5, and was not fully attenuated.

The LMS algorithm under-performed our proposed WLMS algorithm, resulting in lower signal integrity that will negatively affect the classification of CAD. Thus, the following results in this section use the WLMS algorithm with FL = 512 and μ=0.1, which was tuned in this subsection to achieve high attenuation without affecting the lower-frequency content.

### 6.2. Multiple Tones

**Figure 11:**
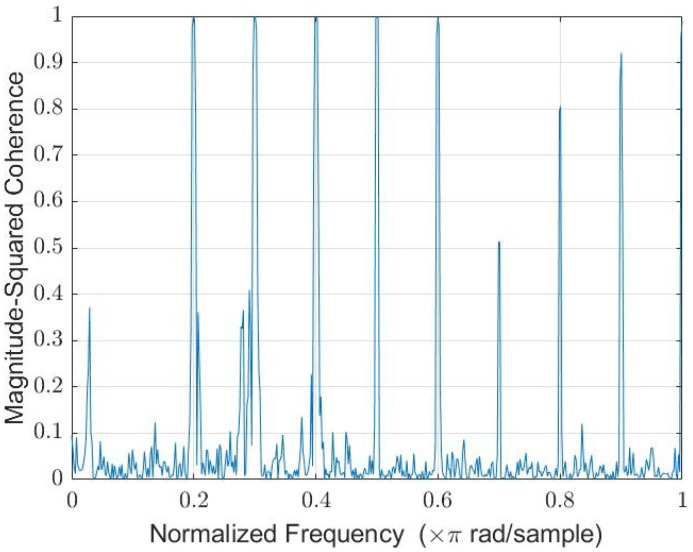
The 200, 300, and 500 Hz tone background noise coherence function via Welch.

The 200 Hz, 300 Hz and 500 Hz tones were played simultaneously through the speaker, and the coherence between the HM and BNM is displayed in Figure 11.

**Figure 12:**
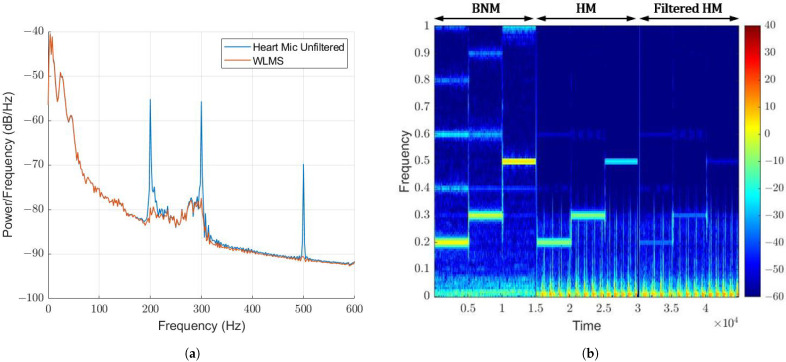
WLMS algorithm on changing tones. FL = 512, μ= 0.1 (**a**) PSD Comparison. (**b**) Spectrogram comparison.

The three tones had unity magnitude-squared coherence, as well as the first harmonic of each tone. This suggests that the first two harmonics can be fully attenuated. The WLMS algorithm was tested on the scenario where each tone was played for 5 s while a heartbeat was recorded, so as to test the ability of the algorithm to adapt to different tones. Near-complete attenuation was observed in the PSD comparison of the filtered and unfiltered HM signal shown in Figure 12a, which is visually seen in the spectrogram shown in Figure 12b. Here, the BNM, HM and filtered HM spectrograms are displayed side by side, where the 200 Hz, 300 Hz and 500 Hz tones were approximately attenuated by 24.5 dB, 21.4 dB and 20.3 dB, respectively.

The 200 Hz tone lies within the signal of interest. It did not affect the heartbeat signal when attenuated, highlighting the strength of the adaptive filtering. The BNM and HM also support the hypothesis of the stethoscope design, where the BNM detects the background noise, and the HM detects the heartbeat plus the background noise. This is essential for adaptive noise cancellation, so as to not affect the signal of interest. The HM did not detect the higher-order harmonics to the same degree as the tones, suggesting the reason unity coherence was not seen in Figure 11.

### 6.3. Hospital/Clinic Background Noise

**Figure 13:**
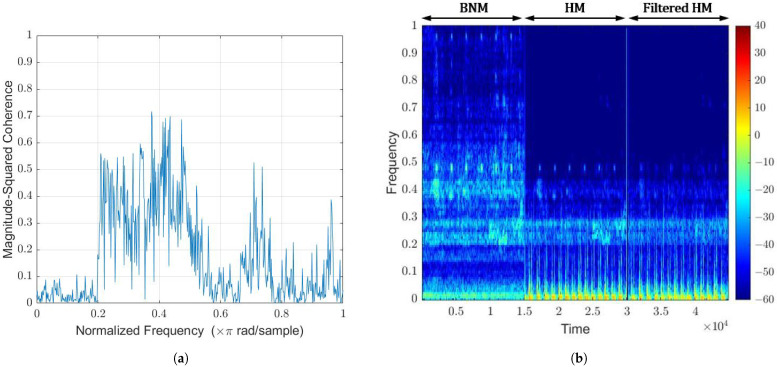
WLMS algorithm on hospital/clinic background noise. (**a**) Coherence function via Welch (**b**) Spectrogram comparison.

This type of noise can be expected when measuring heartbeats in practical situations, such as in hospitals or clinics. Thus, it is of high interest to attenuate this type of non-stationary noise within the frequency of interest. The magnitude-squared coherence between the HM and BNM, shown in Figure 13a, is not in unity for any frequency band. Between 200–500 Hz, it varies from 0.3 to 0.7. Thus, adaptive filtering will not result in the complete attenuation of the background noise. Around 3–5 dB will be possible at a magnitude-squared coherence value of 0.5. There is visual evidence of attenuation displayed in the spectrogram comparison in Figure 13b. The energy at 500 Hz and the band between 200 and 300 Hz is slightly suppressed. To increase the attenuation, applying the WLMS algorithm across separate frequency bands can be explored. A re-plot of the coherence function for each frequency band will provide a better indication of achievable suppression.

### 6.4. Breathing Noise

**Figure 14:**
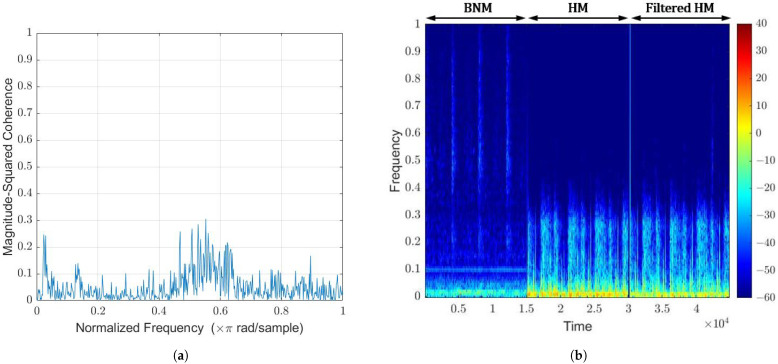
(**a**) Breathing noise coherence function via Welch; (**b**) Spectrogram comparison.

Many studies in the literature on the detection of CAD ensures that patients hold their breath whilst heartbeat measurements are taken [21,22,23]. Although this is ideal for CAD detection, extending the stethoscope system to detect lung disease could be performed in unison, where the breathing noise can contain critical information. Thus, it is of great interest to separate the heartbeat sound from the breathing sound, so they can be studied and processed separately. For this test, the first author breathed with no external background noise. Across the whole frequency band, the magnitude-squared coherence function was less than 0.3, as seen in Figure 14a. This suggests that less than 1 dB of breathing noise suppression is achievable. This was confirmed in Figure 14b, where the HM signal before and after filtering appear identical. Non-linear methods may be explored to suppress the breathing noise and enhance the heart signal. These other methods should be carefully designed, so as to not suppress murmurs that are present in CAD patients, as the diagnostic process will be negatively affected.

The spectrogram comparison between the BNM and HM is worth noting. The BNM has observed power in the phase of the recording corresponding to exhaling. This is not seen in the HM, indicating that both microphones detect the breathing sounds differently. From the HM perspective, the sound generated by the air moves through body tissue and bone from inside the body and is coupled through the diaphragm. In contrast, breathing sounds from one’s mouth and nose travel through the air before being detected by the BNM. Both of these pathways have different acoustic properties, suggesting why the breathing appears differently on each spectrogram, indicating its difficulty to suppress or separate.

### 6.5. Summary of Results

A summary of our results and previously reported results from Section 3 is presented in Table 1.

## 7. Conclusions

In this paper, the performance of the WLMS algorithm to suppress background-noise-corrupting heartbeat measurements was predicted using the coherence function and tested. When periodic signals, such as the single and multiple tones, were played as background noise, the magnitude-squared coherence between the HM and BNM was unity. Thus, we expected that full suppression was attainable and saw this by adjusting the FL and step size, μ, of the WLMS algorithm. Confidence in using this algorithm increased after it outperformed a conventional LMS algorithm on the single-tone background-noise scenario. These parameters were used for the following background noise sources and breathing noise. The magnitude-squared coherence in the hospital/clinic background noise scenario suggested that 3–5 dB noise suppression was achievable using these linear techniques, and this was evident in the spectrogram comparison. For the breathing-noise scenario, the low magnitude-squared coherence resulted in no visible suppression on the spectrogram comparison. To achieve higher attenuation in scenarios where there is low magnitude-squared coherence between the HM and BNM, linear techniques, such as the WLMS algorithm presented in this papers system model, could be applied to separate frequency bands. If this does not achieve desirable suppression, non-linear techniques can be explored.

Our tests produced comparable performance to previously reported data presented in Section 3 and Table 1. Our highest achieved attenuation arose from the scenarios when tones were the background noise source under test. The synthetic chirp and high-frequency noise produced the highest attenuation reported in [9], and the NLMS algorithm led to 20 dB attenuation for 100 dB SPL aircraft noise reported in [10]. Similar to the periodic tones, these sources are stationary, and, although coherence function analysis was not included in these studies, we can assume that there was a high magnitude-squared coherence between both microphones in each case to explain the results. The scenario in [9], where background voices were attenuated by 14.04 dB, is encouraging towards our future studies, as this was considerably higher than the hospital/clinic background noise attenuation that we measured. These are non-stationary sources, indicating our algorithm must be tuned to achieve comparable results.

## Figures and Tables

**Table 1:** Comparison of our results to other studies.

Noise under Test	Study	Algorithm	Measurement	Magnitude Squared Coherence	Attenuation [dB]
Single 300 Hz Tone	Us	WLMS	Heartbeat	1	35 (full)
Single 300 Hz Tone	Us	LMS	Heartbeat	1	32
Multiple Tones: 200 Hz	Us	WLMS	Heartbeat	1	24.5
Multiple Tones: 300 Hz	Us	WLMS	Heartbeat	1	21.4
Multiple Tones: 500 Hz	Us	WLMS	Heartbeat	1	20.3
Hospital/Clinic Background Noise: 200–500 Hz	Us	WLMS	Heartbeat	0.3–0.7	<2
Breathing Noise	Us	WLMS	Heartbeat	<0.3	0
Background voices	[9]	WD	Heartbeat	N/A	14.04
Synthetic chirp	[9]	WD	Heartbeat	N/A	15.5
High Frequency Noise	[9]	WD	Heartbeat	N/A	16.11
100 dB SPL aircraft noise: 100–600 Hz	[10]	LMS	Lung Sounds	N/A	15
100 dB SPL aircraft noise: 450–600 Hz	[10]	NLMS	Lung Sounds	N/A	20

## Data Availability

The data presented in this study are available on request from the corresponding author. The data are not publicly available due to privacy.
